# Animal Leptospirosis in Latin America and the Caribbean Countries: Reported Outbreaks and Literature Review (2002–2014)

**DOI:** 10.3390/ijerph111010770

**Published:** 2014-10-16

**Authors:** Jessica Petrakovsky, Alejandra Bianchi, Helen Fisun, Patricia Nájera-Aguilar, Martha Maria Pereira

**Affiliations:** 1 The National Reference Laboratory of World Organization for Animal Health, National Service of Agrifood Health and Quality (SENASA), Buenos Aires, CP C1063ACD, Argentina; E-Mails: jpetrako@senasa.gov.ar (J.P.); abianchi@senasa.gov.ar (A.B.); 2 Pan American Health Organization (PAHO)/WHO, 525, 23rd Street, N.W. Washington, DC 20037, USA; E-Mails: hfisun@gmail.com (H.F.); najerapa@paho.org (P.N.-A.); 3 Oswaldo Cruz Institute/FIOCRUZ, WHO Collaborating Centre for Leptospirosis, Rio de Janeiro, 21040-900, Brazil

**Keywords:** *Leptospira*, leptospirosis, rodents, dogs, livestock, wild animals, Eco Health, One Health

## Abstract

Leptospirosis is a worldwide zoonotic disease whose transmission is linked through multiple factors in the animal-human-ecosystem interface. The data on leptospirosis reported to the World Organization for Animal Health (OIE) for Latin America and Caribbean (LAC) countries/sovereign territories from 2005–2011 were mapped, showing a wide distribution of outbreaks in the region. Tropical terrestrial biomes are the predominate ecosystems showing reports of outbreaks. Climatic and ecological factors were relevant to the occurrence of epidemic outbreaks. The available scientific information from 2002–2014 was summarized to obtain a general overview and identify key issues related to the One Health approach. The primary serological test used for diagnosis and for conducting surveys was the microscopic agglutination test (MAT). Reports regarding the isolation and typing of leptospires were scattered and limited to data from a few countries, but their results revealed considerable biodiversity at the species and serovar levels. A total of six out of 11 currently named pathogenic species were found in the region. There was also high diversity of animal species showing evidence of infection by leptospires, including rodents, pets, livestock and wild animals. Prevention and control measures for leptospirosis should consider issues of animal and human health in the context of ecosystems, the territorial land borders of countries and trade.

## 1. Introduction

Leptospirosis is a widespread global zoonotic disease with a noteworthy human-animal-ecosystem interface. The bacteria that cause this disease belong to the genus *Leptospira.* This genus comprises 22 species grouped into three categories: pathogenic, intermediate and saprophytic species. Currently, there are more than 250 named, potentially pathogenic serovars [1]. The sources of infection are infected animals [2]. Multiple factors involving animal husbandry, human behavior and climate contribute to the occurrence of epidemic outbreaks in animal or human populations in Latin America and Caribbean (LAC) region [3,4,5,6]. Since this disease is emerging as a public health problem in LAC the assessment of the current situation of leptospirosis in animals could be an important contribution to the technical cooperation in the region under the One Health approach [3].

Different animal species may be carriers of *Leptospira* for long periods. Water or moist soil contaminated with the urine of infected animals is the main vehicle of inter and intra-species transmission [7]. The main transmission routes are through injured skin and via long periods of exposure to contaminated water or soil. The possibilities for transmission through direct contact within animal populations include sexual contact or artificial insemination [7]. The implementation of control measures may be challenging because of the increasing amount contact between domestic animals and wild fauna (mainly rodents and marsupials). The reduction of wildlife habitats favors spillback and spillover processes at the wildlife-livestock-human interface possibly leading to the emergence of leptospirosis in some geographic areas [8,9]. 

Leptospirosis affects virtually all mammals and is characterized by a broad range of clinical symptoms. Domestic and livestock animals can present acute or chronic infections. Clinical signs of acute or sub-acute disease are observed in the leptospiremic phase. Clinical signs related to chronic infections in livestock are usually associated with reproductive losses through abortion and stillbirth [10]. Persistent colonization of the uterus and oviducts may be associated with infertility and stillbirth. Milk drop syndrome occurs in the early stages of infection [11]. Animals that have recovered from the acute phase may develop a carrier status, in which leptospires can remain in the renal tubules for variable periods of time and are excreted in urine. Less serious infections also occur, which may be clinically unapparent but can lead to the development of renal carriers [12]. Infections in goats and sheep can be severe or subclinical and may manifest as reproductive problems such as infertility, abortion, stillbirth and weak lambs/goat kids. The abovementioned aspects of morbidity and lethality in observed livestock indicate the potential for substantial economic losses.

The available information on animal leptospirosis in the Latin America and Caribbean (LAC) region from 2002–2014 was collected and analyzed to obtain an updated overview. The official data analysis was based on country/territory reports to the World Organization for Animal Health (OIE). The available scientific publications generated in LAC were searched, retrieved and analyzed for their content, quality and relevance to supplement the official countries data. 

## 2. Methodology 

*Official Data:* Official data were obtained from the World Animal Health Information Database (WAHID) Interface of the World Organization for Animal Health (OIE). The WAHID Interface provides access to all data held within the World Animal Health Information System (WAHIS). A wide range of information is available from both immediate notifications submitted by member countries reporting exceptional disease events and follow-up reports. Semiannual and annual reports are available covering the diseases listed by the OIE in each member country. Information regarding leptospirosis was available between the period of 2005 to 2011 [13].

*Bibliographic Search:* The following databases were used in the searching and retrieval of published information from 2002 to 2014: PubMed, Web of Science, BVS-BIREME (SciELO, LILACS, Cochrane and WHOLIS), SIDALC (Alliance of Agricultural Information Services for Latin America and the Caribbean), Google Scholar, ISID and ProMed-mail. Bibliographic references were managed using Mendeley software. The keywords *Leptospira* OR Leptospirosis were used as the main subject in all databases. Refinement of the searches was conducted according to the potential functions of each database using complementary words and focusing on animal leptospirosis. Spanish, Portuguese, English and French are the languages spoken in LAC that were considered in the bibliographic searches.

*Bibliographic Analysis and Citation:* All published and peer-reviewed studies with available full text were included. The publications were selected based on the relevance of the information they contained, the methodology applied and the quality of the paper. The following data were considered to be relevant: (1) the incidence of clinical disease in animal populations; (2) data from serological and bacteriological surveys; (3) data regarding serovar circulation. 

## 3. Results and Discussion

### 3.1. Geographical Distribution of Animal Leptospirosis Outbreaks in Latin America and Caribbean (LAC)

**Figure 1:**
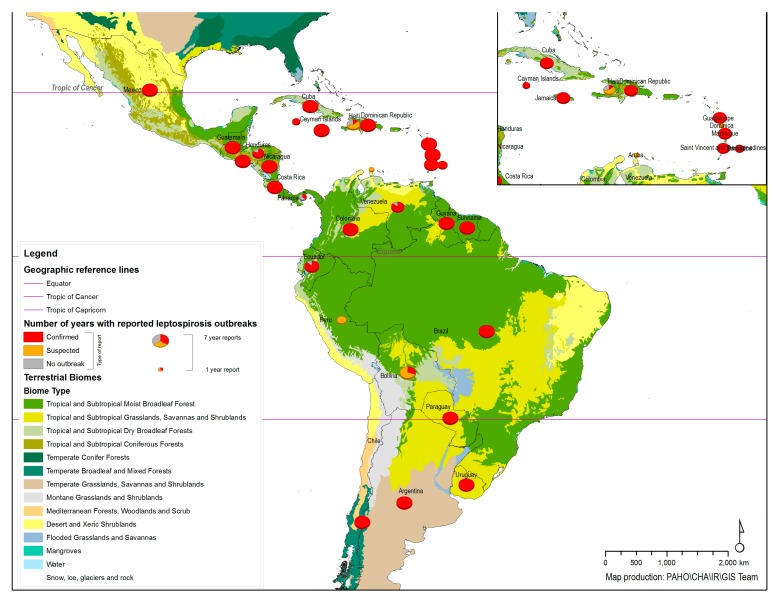
Geographic distribution of animal leptospirosis outbreaks reported to the World Organization for Animal Health (OIE) from 2005–2011.

Figure 1 shows the distribution of leptospirosis outbreaks reported to the OIE from 2005–2011 with the biome types in the background. A wide distribution can be seen in the map, with 27 countries/territories having reported confirmed cases representing outbreaks or foci of the disease. The size of pie chart on the map represents the number of years that countries/territories reported leptospirosis outbreaks during the period, the slice/color represents the type of outbreak report: Confirmed outbreak, Suspected outbreak or No outbreak. The persistence of the disease and regular patterns were observed in the records, without any significant changes in the general pattern during the time period. The countries/territories that have reported the occurrence of the disease in animals have also reported cases of human leptospirosis in the same information system (WAHIS/OIE), except for Belize, which has reported a few cases of human disease but no animal infections.

Tropical terrestrial biomes are the predominate ecosystems showing reports of leptospirosis outbreaks in animals, except for in Chile, which encompasses a remarkable variety of landscapes and major climatic subtypes. Tropical terrestrial biomes are home to a great diversity of animal species that may be the main reservoirs of the bacteria. The weather conditions in these geographic areas certainly favor different mechanisms of transmission between animals and from animals to humans. However, the presence of human cases (if not transmitted from abroad) indicates the presence of animal carriers spreading the bacteria through the surrounding environment. 

The data reported to OIE reflect the existence of diagnostic laboratories and surveillance systems that are ready to detect cases and outbreaks. These laboratories are usually located at reference institutions for public or animal health in the respective countries. It is important to mention that the laboratorial confirmation of cases currently relies on specific tests that are usually performed by reference laboratories linked to the national surveillance systems or research institutions. The unpreparedness of the systems to detect cases implies underreporting or lack of records. It should be also mentioned that the availability and use of simple tests and commercial kits is still very limited for both human or animal leptospirosis cases [2].

### 3.2. Synthesis of the Available Scientific Information About Animal Leptospirosis in Latin America and Caribbean (LAC) 

The indexed and searchable studies focusing on animal leptospirosis during the timeline from 2002 to 2014 involved a wide variety of animal species, sample sizes and geographic areas. This fact represents a limitation regarding the comparability between the results of those studies. The publications were concentrated in only a few countries/territories, which adds an important limitation to the spatial distribution analysis. However, some aspects were considered to be robust across all types of studies, such as the following: (1) a predominance of serological or bacteriological surveys; (2) the wide use of the microscopic agglutination test (MAT), as recommended by the WHO guidelines for leptospirosis diagnosis; (3) sampling and positive results showing statistical significance, generally expressed as percentages based on the number of cases or farms; and (4) expected results regarding the *Leptospira* species or serovars and their common animal hosts, except for wild animals that were found to be carriers of both previously known serovars and new serovars. 

The PubMed search allowed the papers with available full texts to be distributed by country (data not shown). Three countries appeared in a number of papers: Brazil, Mexico and Colombia. One country, Peru, reported the presence of the infection without quantitative data or a confirmed suspicion to the OIE, but there were papers for this country containing relevant information regarding the serovars and disease frequency in the Peruvian Amazon region. Additionally, Argentina had significant information published about animal leptospirosis and has been reporting the disease regularly without quantitative information. Three countries, Costa Rica, Cuba and Uruguay, presented regular reports with quantitative data but did not exhibit any publications focusing on the incidence or the prevalence of animal leptospirosis. 

#### 3.2.1. Leptospirosis Diagnosis: Isolation with Typing, PCR and Serological Tests

The clinical disease or the carrier state can be definitively confirmed through the isolation and identification of *Leptospira*. However, this is not a simple task because: (1) there is only a brief period in which the bacteria can be found in biological samples in the acute phase or requires procedures which can be invasive or lethal in the course of infection; (2) this spirochete grows fastidiously in culture medium; and (3) the complexity of the current identification procedures requires national and international reference laboratories, particularly if it is a new serovar or species. The aforementioned factors explain the small number of publications that record the species and infecting serovars found in LAC. PCR-based tests are used infrequently and mainly for diagnosis in livestock [14,15,16,17]. 

The studies focusing on the isolation and identification of *Leptospira* were found to be concentrated in five countries (Argentina, Brazil, Mexico, Peru and Trinidad and Tobago). The conclusions may not be projected to the entire region, but it should be noted that a total of six species among the 11 known to be pathogenic and 2 currently defined as *intermediate* were recorded (Table 1). There were also isolates that were not identified at the species and serovars levels that remained as unknown pathogens within the genus *Leptospira* [18]. The observed genotypic and phenotypic biodiversity of *Leptospira* was impressive and may be related to the diversity of animal species that can be carriers in this region.

**Table 1:** *Leptospira* species, serovars and genotypes isolated from infected animals in LAC, 2012–2014.

Country	Animal	*Leptospira* spp.
Argentina	Squirrels	*L. interrogans* serovars Icterohaemorrhagiae and Canicola [19].
	Cows and Pigs	*L. interrogans* serovar Pomona [20,22,23].
	South American gray fox (*Lycalopex griseus*)	*L. interrogans*, a new genotype [24].
	Dog fetus	*L. interrogans*, a new serovar designated Baires [25].
Brazil	Marsupials	*L. borgpetersenii* serovar Castellonis [26].
	Capybara (*Hydrochoerus hydrochaeris*)	*L. interrogans* serogroup Icterohaemorrhagiae [27].
	Cattle	*L. interrogans* serovar Canicola and Copenhageni, *L. kirshneri* serovar Grippotyhosa [12,28].
	Swine	*L. interrogans* serovar Canicola [28].
	Sheep	*L. noguchi* serogroup Autumnalis [29].
	Dogs	*L. noguchi* [30]*, L. interrogans* serovar Canicola [28].
Mexico	Cattle	*L. kirshneri* serovar Hardjo [31].
Peru	*Rattus norvegicus* and *Rattus rattus*	*L. licerasiae* serovar Varillal [9].
	Bats	*L. interrogans*, *L. kirshneri*, *L. borgpetersenii* and *L. fainei* [8].
Trinidad and Tobago	Dogs	*L. interrogans* serovar Copenhageni [32].

The conventional MAT was the diagnostic test of choice for serological diagnoses and surveys and served as the gold standard for evaluating new techniques. It was usually performed according to the procedures published in the WHO/ILS guidelines or previously published guidelines with minor changes as reported by authors [2]. There were no descriptions regarding changes in the recommended panels by areas or countries/territories. However, changes in the number or strains employed were observed across the studies. It should be mentioned that the panels of serovars have been distributed to the LAC countries/territories by the WHO Collaborating Centers for quite some time. It is believed that the reference strains may come from the international collections of those centers. Changes in panels most likely arise due to the difficulty of maintaining the panels with all of the reference strains over time. In a few examples, local strains, primarily consisting of new serovars, were added to improve the sensitivity of the technique, as recommended [2]. There were also variations in the applied threshold for positivity, but the reported results were quantitative, due to using serial dilutions twice. A higher titer was frequently used as an indication of the putative infecting serovar. The abovementioned variations can influence the level of the sensitivity and specificity of the technique and hamper comparability across studies. The main interpretations rely on the generic result of a positive or negative sample. 

The results of the MAT can be considered to predict circulating serovars or extrapolated to serogroups in the region. However, the data are indirect and imprecise due to cross-reactions that occur. The criterion of considering the higher titer, which is frequently used by the authors, is not accurate because: (1) at times the higher titer cannot be determined; (2) the differences between the serological titers may be within the margin of error of the dilutions; and (3) there is a certain degree of subjectivity inherent in the MAT. The abovementioned limitations hinder the mapping of serovars found in LAC based on the available serological information. Appropriate information should be based on the isolation and identification of the infecting agent. Additionally, the information about serovars or species from serological or bacteriological studies is limited to a few studies in a few countries.

#### 3.2.2. Animal Species and Herds Infected by Leptospira

A total of 137 articles were reviewed for data extraction and synthesis of the available evidences. The numbers of papers by type or group of animal were: rodents (*n* = 16), dogs (*n* = 25), cattle (*n* = 63), swine (*n* = 10), small ruminants (*n* = 13), camelids (2) and wild animals (*n* = 24). Nine articles had data in more than one of the categories mentioned above. The number of publications is limited considering the extent of the geographical area and the possible implications for animal and human health as well as economic losses. 

Different animal species with and without observable clinical signs were found to present serological or bacteriological evidence of infection. Rodents, dogs, livestock and wild animals constituted part of the recorded mammalian clade that may be potential carriers of leptospires. Positive reactions to a considerable number of different serovars were found using the MAT. High prevalence rates were found in different types of samples from specific populations, herds or groups that were established by the authors [33]. There were also reports of studies in which the animals did not show apparent clinical signs, but the infection was diagnosed by isolation and tissue changes compatible with leptospirosis. In other species, the disease mimics the human infection, as demonstrated via experimental infections in previous studies [34,35]. The most common examples of *Leptospira* isolation from the kidneys without any MAT-detectable antibodies were found in rodents and marsupials [30]. These characteristics were classically described for some species, although the immunological mechanisms involved are not currently understood. The question becomes even more obscure regarding wild animals and new serovars, as found in both previous and new studies within the time period and geographic area of this compilation [30,36,37].

*Rodents:* Synanthropic rodents, primarily the brown rat (*Rattus* *norvegicus*), were found to be carriers of or showed positive serology for *L. interrogans* serovars Icterohaemorrhagiae and Copenhageni [38]. This was expected based on early publications on this topic [2]. Other serovars and serogroups were detected at lower frequencies, such as Autumnalis, Ballum, Canicola and Grippotyhosa, which were mainly associated with other rodent species that are usually found in rural areas or forests [39,40,41,42]. Human disease and infections in dogs were found to be correlated with the presence of rodents in primarily urban areas [43]. A new serovar designated Varillal was described as being associated with rats (*Rattus norvegicus* and *Rattus rattus*) in Peru [9]. Rodents are considered to be universal carriers of *Leptospira*. They harbor the spirochetes in their renal tubules and spread them to the surrounding environment through contaminated urine.

*Dogs:* The retrieved papers regarding leptospirosis in dogs focused on infected animals showing clinical signs and symptoms or evidence of infection based on serology [32,44]. The studies were not comparable in terms of the sample size or representativeness of the populations, which included pet animals, stray dogs and both vaccinated and non-vaccinated hunting dogs. The serological positivity indexes ranged from 4.9% to 73.3% [45,46]. There were serological or bacteriological findings reported from Argentina, Brazil, Chile, Colombia, Peru, Trinidad and Tobago, Chile and Mexico [44,47,48,49]. The isolates from dogs were identified as the *L. interrogans* serovars Canicola, Icterohaemorrhagiae and Copenhageni and the *L. noguchi* serogroup Australis [28,32,50]. Other serovars were predicted according to serology, such as Autumnalis, Bratislava, Grippotyhosa, Hardjo, Louisiana and Mankarso, but they were detected less frequently than Icterohaemorrhagiae, Canicola and Copenhageni [51,52,53,54,55]. Serological data from dogs should be interpreted with caution because the sera from vaccinated dogs may be positive for serovars included in the vaccine. Some studies were conducted in stray dogs and therefore contained no information about prior vaccination. A potentially new serovar designated Baires, belonging to the *L. interrogans* serogroup Djasiman was isolated from an aborted dog fetus in Argentina [25]. The dogs can spread the bacteria via environmental contamination through infected urine. 

*Livestock:* In a broad sense, livestock refers to any breed or population of animals kept by humans for a useful, commercial purpose. This can mean domestic, semi-domestic or captive wild animals. There were only a limited number of studies on livestock, despite their economic importance for the countries in the region. There was a lack of focus on relevant aspects such as the assessment of economic losses in livestock. Health issues in the possible movement of animals across land borders as a result of trade must be evaluated. However, the studies showed that the major etiological agents (species and serovars of *Leptospira*) typically found in livestock throughout the world are present in the region. Below is a summary of the data published according to the animal type or herd. 

*Cattle:* Leptospirosis in cattle may result in significant economic losses due to abortion, embryonic death, the death of calves within the first few days of life and milk drop syndrome. Studies on the prevalence of infection and disease in cattle were concentrated in seven countries (Argentina, Brazil, Colombia, Cuba, Peru, Trinidad and Tobago and Venezuela) [56,57,58,59]. The rate of positivity according to the MAT ranged from 16.4 to 100%. Studies were conducted involving beef cattle and dairy cattle. Low fertility, pregnancy losses and a reduction in milk production were reported [60]. The most frequent serovars predicted by the MAT were Hardjo, Wolfii and Tarassovi [57,61,62,63,64]. The following species and serovars were obtained via isolation from calves: *L. interrogans* serovar Pomona was isolated from dead calves during an outbreak in Argentina [20]; *L. interrogans* serovars Canicola and Copenhageni were isolated from cattle in Brazil [12,28]; and *L. kirshneri* serovar Grippotyhosa and *L. interrogans* serovar Hardjo were isolated in Mexico [31]. The vaccination of cattle could be responsible for the serological findings of serovars present in vaccines, but according to some authors, vaccination was not widely practiced in the examined region [65,66,67]. However, it was also reported that vaccination improved reproductive performance in Brazilian cow-calf systems [68].

*Swine:* The signs of swine leptospirosis are abortion, stillbirth and the birth of weak or ill piglets, appearing 14–60 days after infection [69]. There was a paucity of data and few studies conducted on swine, especially in relation to animal breeding and economic losses. The most frequent serovars that were reactive in the MAT were Icterohaemorrhagiae, Pomona and Tarassovi. Evidence of infection caused by serovar Pomona was associated with stillborn piglets and mummified fetuses, and Icterohaemorrhagiae infection was also related to stillborn piglets [69]. Serological positivity for Castellonis and Icterohaemorrhagiae was reported in Argentina; for Icterohaemorrhagiae, Australis and Ballum in Trinidad; for Bratislava in Jamaica; and for Bratislava, Castellonis and Icterohaemorrhagiae in Mexico and Argentina [56,70,71,72]. The *L. interrogans* serovar Canicola has been isolated from swine [28].

*Small ruminants:* There were a few papers addressing leptospirosis in small ruminants (goats and sheep). In Brazil, the serovars predicted based on MAT positivity in sheep were Bratislava, Castellonis, Copenhageni, Grippotyhosa, Hardjo, Hebdomadis, Icterohaemorrhagiae, Pomona, Sentot, Wolfii, Sejroe and Shermani [14,17,73]. *L. noguchi* was isolated from sheep and reported as an unexpected finding [29]. These authors draw attention to the possibility of widespread infections in herds and farms, though this remains to be quantified. There was an association between the history of infertility and the prevalence of seropositivity in flocks [29,74,75]. A leptospirosis outbreak in goats with reproductive failure was also reported [76]. PCR-based techniques were considered to be valuable tools for identifying the carrier state in sheep and goats [77].

*South-American camelids:* The production of llamas, guanacos and vicuñas has been expanded in the region over the last several decades as an economic alternative. Leptospirosis has been recognized as a cause of abortion in llamas and alpacas. There were two papers presenting the results of serological surveys conducted in camelids from Peru and Argentina. The serovars predicted by the MAT were Pomona, Icterohaemorrhagiae and Canicola in alpacas from Peru and Copenhageni and Castellonis in llamas, guanacos and vicuñas from Argentina [78,79].

##### Wild Animals Showing Evidence of Infection by Leptospira

Table 2 shows the species of wild animals by country that have been found with evidences of infection detected via the isolation of *Leptospira* or positive serological reactions in the MAT. The prevalence studies conducted in wild animals revealed rates ranging from 7.8% to 86.4% in samples of varying sizes. This detailed information is found in the references cited in the same table. There was no accurate representative information regarding entire populations by species and place, other than for captive animals in zoos and rehabilitation centers. The reported aspects related to ecosystems were descriptive and generic. Nevertheless, species from different geographical areas and habitats were found to show evidence of infection, including animals from forest areas, farms and zoological and rehabilitation centers that were geographically distant and in different countries [5,80,81]. Some of the species are native and are found in different geographical areas and habitats (e.g., Amazonian forests, large swamped areas, savannas, mountainous regions) or show a wide geographic distribution in the tropical and sub-tropical Americas (North, Central and South America) [5,79,82,83,84,85]. Additionally, some of the species are considered to be very primitive or endangered [24,85]. It should be noted that wild-captive animals were also reported to exhibit serological evidence or to be carriers of *Leptospira* [82].

The highest rates of positivity were found in the Peruvian Amazon. In this case, the addition of a local strain, recently designated serovar Varillal, to the MAT panel improved the sensitivity and detection level of the technique [86]. The putative serovars based on positive reactions in the MAT were as follows: Andamana, Australis, Autumnalis, Ballum, Bratislava, Brasiliensis, Butembo, Canicola, Castellonis, Copenhageni, Fluminense, Georgia, Grippotyhosa, Hardjo, Hebdomadis, Hurtsbridge, Icterohaemorrhagiae, Javanica, Varillal, Pomona, Pyrogens, Ranarum, Sarmin, Shermani and Wolfii [9,24,85]. The recent reports of a species described as only being found in Peru are noteworthy, raising many questions regarding the processes of deforestation and environmental changes, which impact human and animal health.

**Table 2:** Wild animals showing evidence of *Leptospira* infection by country in Latin America and Caribbean (LAC), 2002–2014.

Country	Wild Animal Species with Evidence of Infection
Argentina	Arboreal squirrels (*Callosciurus erythraeus*), south American gray foxes (*Lycalopex griseus*), wild and domestic carnivores (*Leopardus geoffroyi*), pampas deer (*Ozotoceros bezoarticusceler*) [19,24,87].
Brazil	Non-human primates (*Cebus paella*, *Alouatta caraya*, *Nasua nasua*), gray foxes (*Cerdocyon thous*), rodents (*Dasyprocta s*p.), capybaras (*Hydrochoerus hydrochaeris*), anteaters (*Tamandua tetradactila*), armadillos (*Euphractus sexcintus*), wild canids (*Cerdocyon thous*, *Crysocyon brachyurus*, *Speothos venaticus*, *Pseudalopex vetulus*), raccoons (*Procyon cancrivorous*), white-lipped peccaries (*Tayassu pecari*), collared anteaters (*Tamanduatetradactila*), ocelots (*Leopardus pardalis*), marsupials (*Didelphis albiventris*) and pumas (*Puma concolor*) [83,84,85,88,89].
Colombia	*Rattus rattus*, *Mus musculus*, neotropical primates (*Ateles fusciceps*, *Ateles geoffroyi*, *Cebus albifrons, Cebus paella, Cebus capuccinos* and *Saguinus leucopus*), felines (*Panthera onca*, *Puma concolor*, *Leopardus tigrinus*, *Leopardus pardales)* [90,91].
Peru	Captive collared peccariesRT (*Tayassu tajacu*), capybaras (Hydrochoerus hydrochaeris), *Rattus rattus*, Proechymis, marsupials [18,79,82,83,89,92,93].

### 3.3. Etiological Agents, Hosts and Ecosystems

The data that can be extracted and analyzed from official records and scientific papers show the complexity of natural systems that leads to the occurrence of leptospirosis epidemics in the region. The first aspects to be considered are the biodiversity of the etiologic agent and the great diversity of mammals that can be carriers. Humans are an accidental host. Non-living components, such as water and soil, enable the transport of the bacteria between hosts. The persistence of outbreaks over time and the possible association with tropical terrestrial biomes, as seen on the maps, suggest that certain environmental and climatic conditions favor the possible mechanisms of transmission (Figure 1). However, more information is needed to better understand the multiple causal factors possibly linked to or associated with certain biomes within the geographic extent of the region. The biggest challenges regarding possible control measures are related to the existence of wild foci of infection where the etiological agent can be perpetuated through several different animal species within the mammalian clade.

Although the data available from scientific publications are fragmentary and dispersed in time and space, the presented information about the etiological agent and hosts provides clues leading to key hypotheses regarding epidemiological cycles, including: (1) the presence of universal carriers, mainly synanthropic and wild rodents, that harbor leptospires; (2) infected dogs and other domestic animals; (3) livestock infected with common and uncommon serovars; and (4) wild and wild-captive animals infected with several serovars, including agents exclusively found in this region to date. It should be noted that some findings revealed regional or local peculiarities involving both the host and etiological agents. Some subsets of *Leptospira,* mainly those found in wild animals, may be restricted to biomes or eco-regions. Other serovars found are widespread around the world. There were no studies applying ecosystem approaches, with the exception of a few attempts to correlate some aspects of the interactions between agents, hosts and the environment [48,52,89]. The descriptions of the authors show that the etiological agent and transmission links are present in different ecosystems or biomes, such as pampas, insular environments, mountains, savannas or swamp areas [57,63]. 

Furthermore, considerable biodiversity was observed to be present in the Caribbean Islands, despite the small number of papers published during the period of this search. The most common presumptive serovars were those that are usually found in rodents, domestic animals and livestock [54,70]. Isolates from rodents and dogs have also been reported [32]. A phylogenetic analysis of isolates from humans and rodents in Guadeloupe and Martinique revealed thirteen different genotypes clustered into five main clades that corresponded to the species *L. interrogans, L. kirshneri, L. borgpetersenii, L. noguchi* and *L. santarosai. L. kmetyi* was also recorded as the cause of human disease [94]. The natural history of leptospirosis in island environments can be linked to the history of human occupation of the islands, bringing synanthropic animals, rodents, domestic animals and livestock together.

There were considerable limitations when attempting to associate the serovars found to infect humans and animals due to the lack of integrated information systems. The surveillance systems for human leptospirosis in each country/territory cover large and heterogeneous geographic areas. It is difficult to recognize the disease and receive laboratory confirmation. The underreporting of human cases is higher in rural areas as well, yet cases and outbreaks of animal leptospirosis usually occur in rural areas [95]. Icterohaemorrhagiae and Copenhageni have been isolated from humans, urban rodents and dogs in urban areas. Some serovars identified in domestic animals and livestock have also been found to be a cause of human disease [95]. Little is known about the possible transmission mechanisms involving wild animals and humans. Of particular note are the findings regarding serovars isolated from humans, rodents (urban and rural) and bats in the Peruvian Amazon, where the authors discussed the ecological changes caused by the rate of deforestation in the neotropics with increasing habitat fragmentation and the exposure of humans and domestic animals to the risk of infection by leptospires. Bats are particularly sensitive to anthropogenic activity, but the consequences of such impacts regarding leptospirosis are poorly understood [8].

### 3.4. Key Issues Relevant to Leptospirosis under the One Health Approach

The One Health operational concept has been defined as the collaborative efforts of multiple disciplines working locally, nationally and globally to achieve optimal health of people, animals and the environment. The term is related to the history of Ebola hemorrhagic fever and the threat of avian influenza in 2003. It encompasses diseases whose transmission mechanisms exist at the animal-human-ecosystem interface and that have a negative impact on human and animal health and are related to economic issues. The International Health Regulations (IHR) were implemented to protect public health with the lowest possible influence on international traffic and trade [96]. These guidelines define the criteria for recognizing an emergency of international public health importance. 

Leptospirosis also represents a paradigm of infectious disease in which the man-animal-environment interface has been recognized since ancient times [7]. Multiple factors in complex systems determine the occurrence of epidemic outbreaks in humans or animals. Some aspects observed in the general framework of animal leptospirosis in LAC are similar or are embedded in a vision of global context (ex: frequency of serovars in synanthropic animals, domestic animals and livestock). Others call attention to their peculiarity such as: 1) data on the prevalence of infection in wild animals native to the Andes, the Amazon rainforest and the Atlantic forest; and 2) the records of one species, designated *L. licerasiae*, and two indigenous serovars, designated Varillal and Baires, from Peru and Argentina, respectively. 

Weather-related threats, such those associated with El Niño conditions and episodes, may be associated with epidemics in LAC [6]. This possible association was demonstrated in New Caledonia, where the El Niño Southern Oscillation (ENZO) and meteorological conditions allowed the prediction of outbreaks of human leptospirosis [97]. The official data from countries and publications show common associations between the rainy season, flooding and leptospirosis outbreaks [95,98]. Other environmental variables are associated with the occurrence of outbreaks in humans and animals, as recently demonstrated in a study conducted in Nicaragua [3].

From a global perspective, it has been verified that the disease is widespread and can be considered as emerging or re-emerging, according to the geographical area and time, or as neglected, if observed in a socio-economic and political context. It is estimated that there are over 1,700,000 severe cases of leptospirosis worldwide. The incidence of human disease in the Americas is high, corresponding to an estimate of 12.5 cases per 100,000 inhabitants, compared with a global incidence of 5.1 cases per 100,000 inhabitants [99]. The available information for animals in LAC adds fundamental data on the main sources of infection. However, there are no studies or information systems that integrate human and animal leptospirosis. The relevant policies at the national level are separated into broad sectors related to health, agriculture and the environment, without important integration in terms of information and programs for prevention and control. Therefore, new approaches to generate evidence for guiding the policies and actions of these government sectors are necessary.

## 4. Conclusions

The following topics should be emphasized:

(1) Animal leptospirosis is widely distributed in the LAC region, as demonstrated by the official reports from countries/territories to the OIE and the available scientific publications;
(2) Many mammalian species are potential carriers, including synanthropic rodents, domestic animals, livestock and wild animals;
(3) The different species and serovars of *Leptospira* (isolated or predicted by serological findings in LAC region) represents a big challenge for diagnosis and prevention by using vaccines. Only new and cutting-edge technologies will provide better solutions than the current alternatives;

As leptospirosis could be found in so many species around the region, and considering the existing tools to diagnose and prevent this disease, the elimination is likely impossible. To achieve better efforts in the control measures, the coordination among animal and public health sectors as well as an integrated policy is needed to support actions at the local, national and international levels.
